# Insanity and Demoniacal Possession

**Published:** 1855-10-01

**Authors:** J. May

**Affiliations:** Chaplain to the Hanwell Lunatic Asylum, &c.


### INSANITY AND DEMONIACAL POSSESSION.

BY THE REV. J. MAY, M.A.,

*Chaplain to the Hanwell Lunatic Asylum, &c.*

It has scarcely been a matter of doubt that the Evangelists have recorded several cases of insanity. The best defined are those of the demoniac of Gadara, evidently an example of mania; and the boy brought to our Lord after the transfiguration, whose symptoms closely resemble those of epilepsy. These cases will not fail to bring to the minds of those conversant with the wards of a lunatic asylum, many patients to whom the descriptions might be applied with very little alteration. We observe in these accounts, especially that of St. Mark, a minute attention to details, which would lead us to infer that the writers would not add anything to their statements at all likely to diminish from their clearness and simplicity, or omit to convey an erroneous impression. Yet, in addition to the symptoms of insanity, they mention others which are designated as the effects of what is termed demoniacal possession. Thus, in the two above instances, one is said to have an unclean spirit, the other to have a dumb spirit. Now, regarding these as genuine cases of insanity, it would be a very interesting inquiry, what analogy existed between the insanity of these demoniacs and the simple insanity of our own time? An inquiry, be it observed, which does not necessarily involve or materially affect the much-controverted question of actual demoniacal possession. It was therefore with much disappointment that I found, on reading the article of Mr. Souter in your last number, which he headed with the question, "Does any Analogy exist between Insanity and Demoniacal Possession?" that he had confined the analogy to the title of his paper, and launched out into a few of the arguments usually brought forward by those who deny the supernatural view of this subject. While regretting that this nearly exhausted theme has been so brought forward, I crave permission to insert in your Journal a few strictures upon the arguments which Mr. Souter has adduced in support of his view of this question. Though differing from him *toto cœlo*, I may be permitted to say that his paper is drawn up in a style and spirit which fully bear out the high encomiums which I have heard connected with his ability and character.

There is one point to which I wish to allude before I proceed to criticize the particular argument upon which Mr. Souter has relied. He has made a kind of protest against being considered rationalistic in his views, and I give him full credit for his orthodoxy; but he ought to know that if he adopts the line of reasoning used by the rationalistic writers, and even of the former and worse division of that school, he must not be surprised to be classed with their number. Those who are acquainted with the manner in which these theologians make the language of the Evangelists to mean anything they wish, or nothing at all, will recognise a leaf out of their book in the complacency with which Mr. Souter talks of Eastern languages and personification of influences, not, I am sure, to inculcate infidelity, but to support an opinion. In proof of this, I need only bid any one compare the explanation given by Eichorn and Michaelis of angelic visits, and other supernatural circumstances, with his treatment of this question. For instance, those writers would explain away the salutation of Mary by the angel, by terming it an internal feeling of delight and joy at becoming a mother. Similarly, Mr. Souter says, "that a man is described by the Evangelist as uttering wild words, or as doing the deeds of madness; but the man's mind is not under his own control—an evil influence possesses him; therefore, his deeds and words are not attributed to himself, but to the evil power, or δαίμων." Perhaps an inference the very reverse of Mr. Souter's may be drawn from these words which may be regarded as showing, on the supposition of real possession, that the acts and words of the demon are considered as inseparable from those of the person possessed. Thus the man with an unclean spirit is represented, Mark i. 23, as crying, "Let *us* alone, what have *we* to do with thee, thou Jesus of Nazareth? art thou come to destroy *us? I* know thee who thou art, the Holy one of God." Were the argument a sound one, the fact of its identity with that of the rationalistic writers would not invalidate it; and my object in alluding to this identity is to point out to Mr. Souter, that he must be prepared to carry out this mode of glossing over the literal meaning of the words of the sacred historians to its full extent—a mode of interpretation which, whether in the hand of the former or latter branch of that school, tends to the utter subversion of the Biblical miracles, and, indeed, of the whole Christian system. It is true that he concludes with an appeal respecting the reality and greatness of the miracle of curing insanity, not being affected by the denial of demoniacal possession; but the pious warmth of that appeal must not be suffered to influence our judgment of the question before us; it is ἔξω τοῦ πράγματος. We must not be led by the circumstance that miracles are not denied, to think that this question is therefore not an important one. Truth should be the object of all inquiry, and we must be on our guard lest, finding our main position safe, we should be led by a plausible explanation of a difficulty to yield the outpost, and so admit utter uncertainty into the interpretation of the language of Holy Scripture. These remarks belong rather to Hermeneutics than Psychology; but if plain prosaic statements are not to be construed literally, it is manifestly impossible that we should ever arrive at the truth, and the object of revelation is nullified. Your readers are doubtless well acquainted with Archbishop Whately's "Historic Doubts." I would earnestly recommend the reconsideration of that *brochure* to all who are inclined to tamper with the plain language of Holy Writ.

I now proceed to consider Mr. Souter's argument, and in the first place have to notice the fallacy which it contains. He begins with analogy, &c., then proceeds to show the similarity between the symptons of certain demoniacs and the insane, and thence jumps to the conclusion of their absolute identity, and that demoniacs were merely lunatics. Passing over the want of connexion between the title and the matter of the paper, though I much regret it, I wish to call attention to the strange line of reasoning by which it is attempted to settle this much controverted question. The argument may be thus stated: So and So were demoniacs; but So and So were lunatics; therefore it is concluded, all demoniacs were lunatics; *i.e.*, from two particular and undistributed premises, an universal conclusion is drawn. But there is another fallacy in the argument: no notice is taken of the fact, that even if all demoniacs were lunatics, it would not prove that they were simply lunatics, or that there was no actual possession. Mr. Souter, indeed, speaks very eloquently of our Lord's power over mind being shown by the cure of insanity, as well as over body by the healing of bodily diseases. The cure of lunatics at his word would certainly be an illustration of our Saviour's power over mind, as the healing of bodily diseases was of his power over the bodily frame; but we have no right to insist on the necessity for our Lord's doing anything especially to prove the possession of such a power, which, moreover, is equally manifested by the cure of the insane demoniacs, as it would be by the cure of persons who were merely insane. The sphere of duty in which

Mr. Souter so ably labours, may render the miraculous cure of lunatics peculiarly interesting to him; but the absence of any case of madness, except when connected with demoniacal possession, would not disprove our Lord's power over the minds of men. Nor does the eloquent review of the condition of the Jews at the period of our Lord's sojourn upon earth, as being likely to be productive of many cases of insanity, assist his argument. I am not quite sure, indeed, if such a state of things would be attended by such consequences upon the adult population; it is not the hardened and violent, but the gentle and timid, on whose minds state troubles act generally with the most fatal influence. As in the case of the first French revolution, the terrors of the mothers stamped their dire effect upon their offspring in the form of idiocy or epilepsy; but among grown-up persons, I believe it was not found that any particular increase in the number of insane was produced by the troubles of the time. Doubtless, there were many lunatics when our Saviour was on earth, whatever may have been the cause of their malady; some, at least, of these were brought to Christ, and we are told He cured them. The main cause of their insanity was most likely that which Mr. Souter also mentions, "the unbridled licence of lust and sensuality" In some, this brought on bodily ailments, and led, on their being cured, to such an exhortation as, "Go and sin no more, lest a worse thing happen unto thee;" in others, it was followed by epilepsy, or mental disease, or brought them under the power of Satanic influence, as in the case of the demoniac of Capernaum, who had a spirit of an unclean demon. (St. Luke).

Mr. Souter very properly rejects the summary mode of getting rid of demoniacal possession, by saying that our Lord and his followers described the malady in such terms out of regard to the prejudices of the Jews, and owns that this is neither an adequate nor a fair reply to the difficulty. He then gives us his own view of the matter, that persons afflicted in mind were termed *δαιμονιζόμενοι*, because all evil was the work of the devil. Now I think it will be readily confessed, that whatever opinion the Jews held respecting demoniacal possession, our Lord did not only not undeceive them, but by his words and actions must have corroborated them in their belief. The Jews themselves, we have every reason to believe, considered the possession as a real one. Josephus relates a case of exorcism in which the *δαιμόνιον*, after going out of a man, overturned a vessel which had been placed as a test of his expulsion. This, though probably an instance of legerdemain, shows the state of opinion on the subject. Josephus himself considered that the *δαιμόνια* were the spirits of wicked men, but their actual possession of the demoniacs was not in the least doubted by him. The more common opinion was, that they were wicked angels, *i.e.*, devils. This latter opinion was held by many of the ancient heathen, who believed that there were some bad demons, who had never been men, and who led men into the commission of vice (Plut. Dion.). Xenophon uses the word *δαιμόνια* in the sense of gods, *θεοὶ* (Memorabilia). Cicero regarded the *δαιμονες* as inferior gods, or lares. The earliest Christian writers had seldom occasion to allude to this subject, but when they do so, they take for granted the actual possession. Thus, St. Ignatius, in his answer to Trajan, declares that the heathen gods were really *δαιμόνια*, and speaks as of a matter not questioned of their actual possession of mankind: Οὐδεὶς θεοφόρον ἀποκαλεῖ κακοδαίμονα· ἀφεστήκασι γὰρ ἀπὸ τῶν δούλων τοῦ Θεοῦ τὰ δαιμόνια, and again, τὰ δαιμόνια τῶν ἐθνῶν θεοὺς προσαγορεύεις πλανώμενος· εἷς γὰρ ἔστιν Θεος. But the Jews holding the view of actual demoniacal possession, how can we deny that our Lord was guilty of countenancing their false notion, and confirming them in an error? that He did not foster superstition while professing to proclaim truth? Either demoniacal possession must be received, or our Lord and his apostles must have sanctioned a falsehood. Mr. Souter's explanation, therefore, proceeding, as it does, on the assumption that our Lord's use of the word had a deep meaning, which, as I have shown, the Jews did not attach to the term δαιμονιζόμενοι, falls to the ground. But do the instances which are adduced really support this view, if we leave out of consideration the opinion of the Jews? We gather, indeed, from Holy Scripture the general fact that all evil is the work of Satan, and the consequence of the entrance of sin into the world: but our Lord healed many persons of various bodily diseases, and I think also of mental disease, whom the Evangelists do not say were POSSESSED. On the other hand, we find that this was predicated of certain persons who had mental disease, and certainly of one person also who had only bodily ailment: of this last, the poor woman who had been ill eighteen years, our Lord said that Satan had bound her; but what was in reality her case? She had πνεῦμα ἀσθενείας (so πνεῦμα πύθωνος, Acts xvi. 16), an evil spirit which afflicted her with bodily distemper. There is no doubt as to the synonymous use of πνεῦμα and δαιμόνιον; indeed, St. Luke says that the epileptic boy had a πνεῦμα, St. Matthew that he had δαιμόνιον: and in the same verse δαιμόνιον and πνεῦμα ἀκάθαρτον are both used by St. Luke to express the same evil spirit. It is, moreover, not unlikely that the secret sins of the boy had subjected him to the affliction (ἀκάθαρτον) with which he was visited. In examining this last case, a distinction will be observed between casting out the πνεῦμα, and the cure of the epilepsy. St. Matthew says, that at the rebuke of Jesus the demon departed out of the boy, and that "the child was cured from that very hour." St. Luke says, "Jesus rebuked the unclean spirit, and healed the child." A marked distinction is also made, in the accurate language of the Evangelists, between those diseases which are connected with physical causes, and those which are the consequence of Satanic influence: on the one hand, we have cases recorded of persons suffering from organic disease, *e.g.*, were deaf or dumb (Mark viii. 31, &c.); on the other hand, we meet with similar afflictions connected with demoniacal influence; thus, Matthew (ix. 32) makes mention of a man who was dumb, and again (xii. 22), of one who was both blind and dumb, both of whom he states to have been possessed; and further, it is to be observed, that neither these demoniacs, nor the θυγατὴρ 'Αβραάμ, were insane.

Mr. Souter's explanation is, that by δαιμόνιον is not meant a spiritual being, but only an influence; and in support of this view he asserts that we have no instance recorded of the possession of a person by Satan, the actual διάβολος, but only by δαιμόνες, or influences proceeding from the Prince of Evil. This view cannot be sustained, inasmuch as by δαίμονες were always understood both by Jews and Christians, and also heathens — an opinion confirmed by our Lord and His apostles—real spiritual beings, and it is not supposed that Satan himself, for he is not omnipresent, but his emissaries, were the agents who caused the affliction which we are discussing. But further, it is evident that the Jews thought it possible for Beelzebub, or the Prince of the devils, to possess a man, and our Lord's remarks accorded with their view (Mark iii.); and we not only learn that Satan and Beelzebub were names of the Prince of Evil, but also, that to be possessed by Beelzebub was to be possessed by a δαιμόνιον, or πνεῦμα ἀκάθαρτον (Mark iii., 22, 23, compared with verse 30). The fact seems to be, διαβολος and Σατανᾶς are used to designate the powers of evil under their leader or head, while δαιμόνια, and sometimes δαίμονες, and πνεύματα ἀκάθαρτα, and πνεύματα τῆς πονηρίας denote the subordinate spirits or ἄγγελοι τοῦ διαβόλου (Matthew, xxv. 41). And here I may draw attention to the propriety of Christ's casting out devils; for the Jews would certainly have asserted that His other miracles which attested His divine mission and authority, and the reality of which they could not deny, were wrought by diabolical instrumentality: but the fact of His casting out devils, and so destroying Satan's power, was a complete refutation of that objection. The question, "How can Satan cast out Satan?" was a *reductio ad absurdum* to which they could offer no reply.

I do not attach much importance to the distinction made between the *δαιμονιζόμενοι*, and the *σεληνιαζόμενοι* in Matthew iv., 24, independently of other considerations, but the wording of the whole passage is worthy of notice: in the first place (verse 23), the Evangelist distinguishes between *νόσος* and *μαλακία*, *i.e.*, between sthenic and asthenic diseases; he afterwards speaks of *βασανοι*, *i.e.*, diseases attended with great pain. He then mentions three classes of afflicted persons, the *δαιμονιζόμενοι*, *σεληνιαζόμενοι*, and *παραλυτικοὶ*. Now, we must allow a distinction between the demoniacs and the lunatics, as well as between the lunatics and the palsied; and, in fact, we gather from the passage, that some persons who had bodily ailments, others who were insane or epileptic, and others also who were possessed with devils, were healed by our Lord. From other passages we learn, that some of these demoniacs had likewise bodily diseases, and others of them were afflicted in mind, or mad. On this last point I can add nothing to what Mr. Souter has said; he has ably demonstrated the identity between some of the actions of certain demoniacs and our own insane. But this is all that his paper proves; it does not show that all demoniacs were insane, much less that they were simply insane. Many of the actions attributed to them correspond to those of maniacs, &c.; but there are other circumstances mentioned which completely distinguish them from mere madmen. Their supernatural acquaintance with the person of Christ, their avowal of their wretched condition, &c., cannot be explained on the supposition of mere insanity. The Gadarene demoniac was doubtless a maniac, but as evidently, if language has any fixed meaning, possessed with an evil spirit or spirits. This case affords, perhaps, the most remarkable proof of the reality of possession. The ejected devils entered into a herd of swine, which, in consequence, ran violently down a steep place into the sea. Now, whatever we may attribute to the force of imagination, as explanatory of demoniacal possession, nothing of the kind could have actuated the irrational animals. Shall we say that evil influences went out of the man, or a kind of material madness, like an electric current, went out of the possessed and entered into the swine. How, on this mode of interpretation, shall we understand those words of St. James (iii. 19), *τὰ δαιμόνια πιστεύουσι καὶ φρίσσουσι*? I think no one would be satisfied to translate the Apostle's enthymem, "The evil influences believe and tremble."

Mr. Souter lays great stress upon the charges made by the Jews against our Lord: "Thou hast a devil," and their assertion concerning Him, "He has a devil and is mad." Respecting the former he says, "Our Lord's language appeared to them incoherent raving." Now this is a very different account from that which the Evangelists give of the effects of his discourses. On one occasion it was said, He "spake as never man spake;" on another we are told, that the Jews could not answer His arguments; on another, that He spake with authority, and not as the scribes, *i.e.*, he taught with inherent wisdom, not by borrowed references; and on many occasions the wisdom which He manifested elicited their admiration, as well as put to silence all who ventured to dispute with Him. It was to be expected that his enemies should resort to the expedient of saying that he had a devil, for they thus endeavoured to account for the superhuman power of His arguments; but to attribute to him language merely irrational, or the acts of a merely raving maniac, would have carried with it its own refutation.

I have still to examine the assertion, "*δαιμόνιον ἔχει καὶ μαίνεται.*" So far from this passage proving the identity of the expressions, demoniac and madman, if *δαιμόνιον ἔχειν* means merely *μαίνεσθαι*, with what absurd tautology is the writer chargeable who frequently uses both words? The conjunction *καὶ* distinguishes between the two terms, and also shows that the latter is an addition to, or an effect of, the other (see Passow at *καὶ*). The Jews thought the madness was the consequence of our Lord's having a devil; his friends, who also considered his mind affected, merely said *ὅτι ἐξέστη*, for they did not suppose him to be possessed. The disciples similarly said of Rhoda, Μαίνῃ (Acts xii. 15), but it would manifestly have been absurd to have said to her δαιμόνιον ἔχεις; so also when Festus said to St. Paul Μαίνῃ, there is nothing to connect it with possession; nor did the Apostle in his reply make any allusion to such a condition, (see also 1 Cor. xiv. 23, and 2 Cor. v. 13, where ἐξίστημι is used as the opposite of σωφρόνειν). The state of the case is this: the Jews, who had frequently told our Lord Himself that He had a devil, being anxious to dissuade the people from listening to His discourses, said to them δαιμόνιον ἔχει καὶ μαίνεται, *i.e.*, "He has an evil spirit which has produced madness, therefore it is not worth while to pay attention to such an one."

The signification of the word δαιμόνιον in the Septuagint version, alluded to by Mr. Souter, appears to me to be of some importance in reference to this question: but I submit that there it is *always* used in reference to spiritual beings, and not to mere influences, and gives the exact meaning of the Hebrew phrase. Thus Ps. cvi. 37, (Sep. Ps. cv. 37), לַשֵּׁדִים is rendered δαιμονίοις, and so also Deut. xxxii. 17, "They sacrificed to devils, and not to God." These passages exactly correspond to St. Paul's words, 1 Cor. x. 20. It is indeed true that on one occasion Ps. xcvi., (Sep. xcv. 5) the word δαιμόνια is used as the equivalent of אֱלִילִים or idols: but we shall see that its use on this occasion is exceedingly appropriate, and gives the spirit of the Hebrew text. The literal meaning of the word אֱלִיל is something *false, valueless, nothing worth* as a dependence, and hence appositely idols. It is either formed from אל *nothing* or *no*, by reduplication of the ל to express intensity, or compounded, as some Rabbinical scholars have thought of אל and אֵל no God, *i.e.* false God, so 2 Chron. xiii. 9, idols are called לֹא אֱלֹהִים. When not referring directly to idols, the word אֱלִיל signifies worthless, but has still a reference to them. Thus, Job xiii. 4, "Ye are physicians of no value, רֹפְאֵי אֱלִל, *i.e.* physicians of false Gods: an idiomatic expression corresponding to the addition in Hebrew of God, for what is excellent and great. When nothing, in the sense of *ne quid* is intended, the word used is, בְּלִימָה. Thus Job xxvi. 7, "God hangeth the earth upon *nothing*," the Sep. has ἐπὶ οὐδενός. When אֱלִיל refers merely to the images before which worship was offered, the Septuagint has εἴδωλον, or an equivalent; but in Ps. xcvi. 5, where the word does not refer to images, but to the beings themselves to whom the service was rendered, they have correctly translated by δαιμόνια: "All the Gods of the heathen are devils." This is in accordance with the statement of St. Paul, who says, 2 Cor. viii. 4, "We know that an idol is nothing in the world," but adds, chap. x. 20, "The things which the Gentiles sacrifice, they sacrifice to devils, and not to God; and I would not that ye should have fellowship with devils." In the former place he shows the falseness of the heathen mythological divinities, such as Jupiter, Mars, Venus, as not being *in rerum naturâ*: in the latter he was warning Christians against participation in idol feasts, for by so doing they entered into fellowship with δαιμόνια, *i.e.* the powers of the kingdom of darkness. If δαιμόνια meant merely evil influences, I am at a loss to know how communion or fellowship could be held with them by mankind.

I have thus endeavoured to show the weakness of the arguments used by Mr. Souter to disprove the tenet of actual demoniacal possession. I do not enter directly upon the evidence of the common view of the matter: this being founded on the plain and literal understanding of the language of Holy Scripture, the onus probandi rests with those who deny it. Until this can be done (and although attempted by some of the most able and learned writers, it has been found impossible to do so, except by upturning at the same time the whole fabric of Christian truth), I am bound to believe that evil spirits actually *possessed* men about the time our Lord was upon earth. How or why such power was given to the powers of darkness is another question. What Mr. Souter has really shown, the conclusion which truly follows from his argument, is, that certain demoniacs were at the same time insane, and from this point commences the inquiry, "Is there any analogy between demoniacal insanity and ordinary insanity?"

II. I propose now to throw out a few hints on the manner of conducting this inquiry, and its probable results.

The first point which comes before us is, whether there is demoniacal possession in the present day. I am told that some eminent persons are of opinion that there is. The learned German commentator Olshausen, who himself considered that such influences were no longer exerted by the powers of darkness, nevertheless writes, "Yet it cannot be overlooked that many distinguished physicians, such for instance as Esquirol of Paris, are of the contrary opinion." He also refers to other authorities. I have not, however, been able to trace this opinion in Esquirol's work on Insanity. On the contrary, he appears to me not only to deny the reality of demoniacal possession, but to confound it with demonomania. He also says, "*Si c'en etait ici le lieu, je prouverais que l'on s'est servi des alienés pour rendre les oracles.*" I quite agree with him, however, in considering as monomaniacs the sorcerers and witches of later ages, when they were not impostors; but I submit that there is no resemblance between those wretched beings and demoniacs: they were insane, and often endured the most cruel torments, as they thought justly, because they confessed before their judges that they had intercourse with the unseen world, or possessed magical powers; charges which they were no more able to deny, though false, than the insane in our asylums to conceal the delusions which they entertain.* But of this monomania I propose hereafter to speak.

My own opinion I may state to be, that simultaneously with the cessation of the counterbalancing miraculous powers in the church, the power of Satan was restrained, and that the temptations and subjection which he exerts over many persons (2 Tim. ii. 26) are of the nature of ordinary influences, corresponding to the ordinary influences of the Holy Spirit, as distinguished from his miraculous or extraordinary gifts. Our investigation would be therefore reduced to the inquiry, whether the insane are ever affected by such Satanic influences in a manner at all analogous to the insane-demoniacs of the New Testament times?

In conducting this inquiry, I should at once dismiss all cases of pretension to present supernatural influences: such for instance as those of the heathen priests of Ceylon, the East Indies, Africa, &c., who, when consulted by their votaries, throw themselves into a species of ecstasy by violent exercise. Such modern inspirations I consider as traditionary superstitions derived from the heathen oracles before the Christian era, imitations of the real demoniacal possession of the Pythoness. For although I believe, with Fontenelle, that Christianity has shut up the heathen oracles, it is because Satan's power is now restrained, not because they were never real divinations. The Pythoness dispossessed by St. Paul was a real soothsayer; and I may use, respecting those heathen oracles, the words of the great Roman orator, "Numquam illud oraculum Delphis tam celebre et tam clarum fuisset, neque tantis donis refertum omnium populorum atque regum, nisi omnis ætas illorum oraculorum veritatem esset experta."—(Cic. de div. I.)

I would also pass by all cases of demonomania as merely presenting particular forms of delusions, which differ very little from others which are of a like

* I am acquainted with an elderly female who is anxious that some one should cut her in pieces, because she thinks she has no soul, and that her body is only a phantom filled with wind.

subjective character; nor is the case altered when the delusion is connected with some organic defect, though a *primâ facie* view seems to show in these persons a resemblance to demoniacs, who were usually affected with disease. Thus, one poor woman who had an uneasiness in her throat, and difficulty in swallowing, fancied that Satan was in her; another, who died from a cancer, imagined that the distressing pains which she suffered were caused by devils; but these were manifestly mere forms of delusion, having no real resemblance to thecases of demoniacal possession.

I should also be disposed to omit from consideration many instances in which a totally different character was evinced by the insane from that which appeared natural to them. Some of these it has been thought could only be accounted for by the supposition of Satanic influence. A remarkable instance of this was once described to me by the person who had charge of the lunatic: a young female had been educated in a manner most likely to separate her from the very approach of immorality, having become insane uttered language of the most vile description, and spoke of scenes and actions too horribly obscene to be repeated; and the question was put to me, how could this patient ever have become acquainted with such language and such subjects, unless they were put into her mind by the great enemy of souls? Other similar cases might be mentioned, but I think this knowledge may be otherwise accounted for; *nec deus intersit, nisi*, &c., is a very useful rule: we ought not to turn to supernatural until we have exhausted natural sources of explanation. In the above case, it is not improbable that the evil was learned from domestics, or from books which they had inadvertently left within her reach; but I do think that such knowledge of evil, however acquired, may be used by Satan to harass and distress the mind, and that the admission, so to speak, within the unhallowed precincts of vice has been a source of temptation and evil suggestions, which in vain the voice of conscience has striven to check, till the troubled mind, having become too confused to distinguish between the temptation and the sin, yet horror-struck at the bare idea of the possibility of its commission, has given way in an unequal conflict, into which it had been originally led by the secret knowledge of evil.

So many persons of eminent virtue and piety have been struck down by insanity, that it will be evident that either there were no virtuous persons similarly affected among the Jews, or the word demoniac did not apply to all who were then insane. The insanity of the Cowpers and Halls, &c., so touchingly alluded to in Mr. Souter's paper, were ordinary visitations to which, like bodily diseases, all are liable who pass through this world of trial. Such cases, whatever may be the particular character of the malady, are by their very nature excluded from our inquiry.

I now proceed to show more directly the nature of the investigation, and with this view will recapitulate the most marked features of the New Testament demoniacs, and intimate what are the cases which furnish the analogy among the insane.

1. The first characteristic of the demoniacs was a superhuman knowledge.*

* Olshausen notices a resemblance between mesmeric clairvoyance and this knowledge of the possessed, and that the effect upon the nervous system is very similar in both cases. It is at present difficult to say whether those who run after spirit-rappings, &c., are the dupes of a clever deception, or the victims of their own curiosity. That insanity may be the consequence of such practices, I have no doubt; nay, the effect upon some persons who have attended meetings for calling up the dead, leads one to fear the worst consequences in the more nervous temperaments. Who shall say whether the end may not be demoniacal possession as well as insanity? Esquirol notices the epidemic character of such delusions or practices:—"Demonomanie est quelquefois epidémiques: comme toutes les dealadies nerveuses, elle se propage par une sorte de contagion morale et par la force m l'imitation."

This was evinced on many occasions by their recognition of our Lord and his Apostles, and the object of their teaching, and by divining or soothsaying. Their testimony to our Saviour's person was quite distinct from that given on various occasions by His disciples. The demoniac was rebuked, for Jesus would not receive his testimony; while the convinced disciple was pronounced blessed (Mark i. 24, 25, Matt. xvi. 16, 17). So St. Paul rebuked the Pythoness (Acts xvi. 17, 18).* Of course, on the supposition that there is not at present actual possession, we shall not expect to meet with anything analogous to this superhuman knowledge in the insane.

2. The next characteristic was a moral uncleanness, ἀκαθαρσία, implied in the epithet ἀκάθαρτον, so often conjoined with πνεῦμα. I am quite of the opinion of Olshausen, that the δαιμονιζόμενοι do not appear to be persons who had surrendered themselves up entirely with their whole internal life to sin, but those whose passions had burst the restraints which were imposed by light and knowledge and the dictates of a better will. The πονηρὸς, or wicked man, who had suffered evil to gain possession of his heart without resistance, whose conscience was itself seared or dead, was indeed under the dominion of Satan, but does not appear to have borne the character of the demoniac. This latter manifested a struggle with the evil which he could not shake off, but this very conflict within proved that there still existed a germ of life from which might spring the flower of faith. This better will hurried the poor Gadarene into the presence of Jesus, and caused him to fall down at His feet and worship Him, while the influence of the demoniacal agency was evinced by the cry of terror, "What have I to do with thee, Jesus thou son of God? art thou come to torment us, and cast us into the abyss *of hell* before the time?"

I think there will be found among the insane many who still thus *meliora probant*, but *deteriora sequuntur*. These, it is probable, having first been led astray by temptation, were affected with shame and remorse; or having been guilty of habits of secret sin, which they had not strength to resist, but which their conscience condemned, were goaded to despair and insanity. We do find indeed among the insane also some of those who have drained the cup of iniquity to the very dregs, the ὁι πονηροὶ; it was, however, when the body had been exhausted by excess, or when ruin, the consequence of extravagance, had stared them in the face, and reflection only brought self-condemnation, that the bitterness of remorse seized on the debilitated faculties. But it is not among such cases we should expect to meet the analogy in question: in them, previously to actual remorse, there had been no internal conflict, whereas in the demoniacs there was a struggle between the principles of good and evil. In a Judas, or, to borrow Mr. Trench's illustration, a Klytemnestra, we have the obduracy of the πονηρὸς; while in the conscience-smitten Orestes, tormented by the dogs of hell into madness, the insane demoniac is aptly depicted.

3. Closely connected with the last mentioned characteristic and its physical consequence was some disease, the result of nervous debility which usually showed itself in the form of mania, epilepsy, or a palsied state of the organs of speech, hearing, &c. This last seems to have been a suspension of the use rather than a lesion or disease of the organs themselves. Such seems to have been the case of the epileptic boy; he had a species of convulsion which was a frequent accompaniment of the possession. I would wish carefully to avoid giving an opinion upon the medical view of this subject, but I may be allowed to state, that cases which prove the connexion between certain excesses and the above named diseases are alas! too common. It was not long since I met with a young man stricken down by melancholia, whose sinful habits as he afterwards owned, had been indulged in defiance of the rebukes of conscience, who

* The language of the Gadarene demoniac evinced the same knowledge. The demons besought our Lord not to command them to go ἐις τὴν ἄβυσσον, *i.e.* not the sea, but γέεννα, the bottomless pit.

suffered subsequently to the removal of the melancholic symptoms from a kind of convulsive attacks very similar to epilepsy. He recovered, and is now quite well. This youth's case, and one or two others which originated in the same habit, strongly impressed me as bearing a very close resemblance to that of the boy who had the *τινεῦμα ἀκάθαρτον*.

4. The last feature in the character of the demoniacs to which I shall refer was a kind of double consciousness—a twofold self; the powers of darkness appear to have acted through the human intellect, but not so as at all times to destroy the personal consciousness; it has been described as the action of two souls on one mind. I would compare it with the miraculous influences of the Holy Spirit in the earliest ages of the Church, when the human intellect was supernaturally enlightened and guided for Divine purposes, though its individuality and personality remained. Many writers besides Dr. Guislain, whose remarks are quoted by Mr. Souter, have noticed a similar condition in the insane, and that it is sometimes remembered by the patient when the paroxysm has passed away. Mr. Trench, in his work on Miracles, quotes a testimony to this fact from a German Rationalist, who states that he had been told by an authority of a most unexceptionable character, a person of a cool and mathematical style of intellect, "that it had been satisfactorily proved to the highest Medical Board of Wurtemburg, that there are maladies in which the person has two *consciousnesses*, so that he is convinced that besides himself a second has forced himself into him."* "Patients often, in more lucid intervals, have said that they were urged on by a second self even more powerful than their true self, to the commission of acts which they knew to be wrong, and utterly abhorred." This admits of elucidation, though it cannot be easily accounted for. In sane persons, temptation acting on a free mind is either *ab extra*, or else it rises up in the thoughts in such a manner as not to be distinguished from the succession of ideas which spring up during reflection or meditation, and appear to be the man's own. In the insane, the impulses seem often to be more separately felt. In the former case, the temptation may be compared to forces in composition, which act only by a single impulse; in the latter, it resembles the same forces resolved, when each acts separately, and the evil is seen to overpower or annihilate the better principle. A fourth characteristic is thus furnished to aid us in comparing analogically possession and insanity: as once men became victims of the powers of darkness by their own acts, by rejecting the monitions of conscience, and though offering some resistance, yet yielding to the evil, and of these some also became maniacs, &c., through the indulgence of habits by which their nervous system was weakened; so we shall probably find that similar conduct still brings on similar forms of mental disease, and also that the power of Satan's temptations is influential over such persons notwithstanding that they are insane.

Notwithstanding the great length of this communication, I will beg permission to add a few brief remarks on what I should expect to be the results of such an inquiry.

1. We should frequently find in the insane a struggle going on between good and evil, as it does in the sane, though the state of the mind and the nervous condition render the resistance of the former to the evil very feeble; and we might learn how far an exhibition of Divine truth was capable of assisting the patient to regain a moral self-command, even if it did not tend to the recovery of the mental health.

2. We should also probably discover that the paroxysms of mania to which

* A doubling of the objects of vision was often alluded to by the ancients. Does this refer to the consciousness? Was this the poet's intention, when, describing the melancholic Dido, he says:

> "Eumenidum veluti demens videt agmina Pentheus,

> Et solem geminum, et duplices se ostendere Thebas"?

certain chronic patients are liable, are not unfrequently consequent upon some vicious habit, or the indulgence of a train of thought which draws close to the confines of sin. This knowledge, together with the fact that patients are often conscious at the time, and remember afterwards what they did during a period of excitement, though unable then to still the tempest that raged within them, may perhaps be suggestive of a moral treatment when the paroxysm has passed off. As in the case of the epileptic, whose cure was only to be obtained by prayer and fasting, may not the patient be led, when free from excitement, to seek strength to resist those causes of irritation to the mind or nervous system which upturn every remnant of mental and moral control?

3. Such an investigation may also help to throw some light on the difficult subject of the responsibility of the insane, by exhibiting the twofold influences which probably are at work within them, and by combining the knowledge they possess of the nature of any particular act with the power of resisting the temptation to its commission, we may be led to some more satisfactory mode of judging of the guilt of criminal lunatics than we yet seem to possess.

4. And lastly, we may perhaps learn how, in the conduct of education, to prevent the occurrence of this dreadful malady by instilling those habits of mental and physical restraint, which will subject passion to reason, and the impulses of desire to the pure dictates of an enlightened conscience.

---

